# Admission point-of-care D-dimer for identifying haemostatic envenomation after snakebite: A prospective observational study

**DOI:** 10.1016/j.toxcx.2026.100266

**Published:** 2026-09-28

**Authors:** Deo Mathew, Siju V. Abraham, Sarah Paul, Aboobacker Mohamed Rafi, Appu Suseel, C.K. Kassyap

**Affiliations:** a Department of Emergency Medicine, Jubilee Mission Medical College and Research Institute (JMMC&RI), Thrissur, Kerala, India; b Centre for Dental Education and Research, All India Institute of Medical Sciences, New Delhi, India; c Department of Transfusion Medicine, JMMC&RI, Thrissur, Kerala, India

**Keywords:** Snakebite, D-dimer, Haemostatic envenomation, Venom-induced consumption coagulopathy, Diagnostic accuracy, Biomarker, Emergency medicine

## Abstract

Haemostatic envenomation (HE) — comprising venom-induced coagulopathy, thrombocytopenia, and systemic bleeding — is the principal determinant of snakebite morbidity in South Asia. Conventional coagulation tests lack sensitivity for early haemostatic activation; D-dimer has been proposed as a rapid triage biomarker, but data from Indian emergency settings are lacking. We conducted a prospective diagnostic accuracy study (JMMC&RI, Thrissur, Kerala; March–December 2022; n = 96 consecutive snakebite patients). The primary aim was to evaluate the diagnostic accuracy of admission point of care D-dimer for identifying HE. Secondary aims were to compare its performance with standard coagulation tests and to evaluate it against the broader composite outcome — clinically significant envenomation (CSE). HE was defined as modified Lee–White clotting time (MLW) ≥15 min, failed 20-min whole-blood clotting test (20WBCT), INR ≥1.4, fibrinogen <100 mg/dL, platelet count <100 × 10^9^/L, or systemic haemotoxic bleeding. Comparators were MLW/20WBCT, prothrombin time, INR, aPTT, fibrinogen, and platelet count.

Thirty-three patients (34.4%) met HE criteria. At the Youden-optimal threshold of 620 ng/mL, D-dimer achieved sensitivity 87.9% (95% CI: 72.7–95.2%), specificity 79.0% (67.4–87.3%), PPV 69.0% (54.0–80.9%), NPV 92.5% (82.1–97.0%), LR+ 4.19, LR− 0.153, and accuracy 82.1% (73.2–88.5%). AUC was 0.874 (0.791–0.957) — highest among all comparators: MLW 0.763, WBCT 0.652, aPTT 0.544, fibrinogen 0.728, platelet count 0.681, INR 0.646, and PT 0.615. D-dimer ≥620 ng/mL was the strongest independent predictor of HE (OR 22.04; 95% CI: 6.13–79.25; p < 0.001). Three of four patients with delayed coagulopathy had D-dimer ≥620 ng/mL at admission. Against the broader clinically significant envenomation (CSE) endpoint (n = 52, 54.2%), AUC was 0.885 (0.816–0.954) at 139 ng/mL (sensitivity 88.2%, specificity 81.8%).

Admission D-dimer demonstrated higher discriminatory performance for HE than all standard coagulation comparators. Its NPV of 92.5% supports potential utility as a rapid rule-out biomarker. These findings are exploratory and require external validation before clinical implementation.

## Highlights

• Admission D-dimer identifies haemostatic snakebite envenomation (AUC 0.874)
• Youden-optimal cut-off 620 ng/mL; NPV 92.5% supports rapid rule-out utility
• D-dimer Outperforms MLW, fibrinogen, platelet count, PT, INR, and aPTT
• D-dimer elevated before other bedside clotting tests became abnormal
• D-dimer signal driven by subclinical haemostatic activation in haemotoxic species

## Graphical abstract

**Figure:**
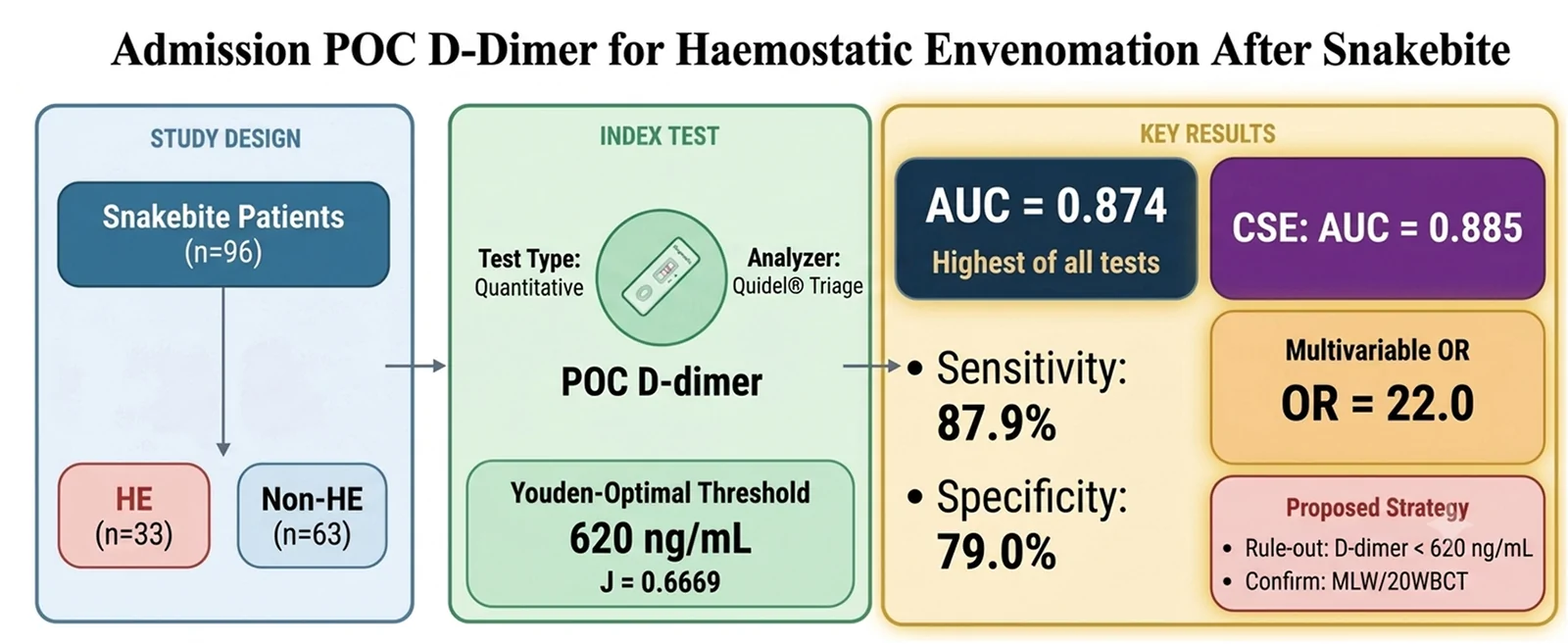

## 1 Introduction

Snakebite envenomation remains a neglected tropical disease of major public health importance, causing an estimated 1.8 million envenomations and 94,000 deaths annually worldwide, with the majority of the burden concentrated in South and Southeast Asia(Kasturiratne et al., 2008). India alone accounts for approximately 58,000 snakebite deaths per year (Suraweera et al., 2020). In peninsular India, Russell's viper (*Daboia russelli*i) and hump-nosed pit viper (*Hypnale hypnale*, HNPV) together account for the predominant burden of haemotoxic morbidity, mediated principally through venom-induced consumption coagulopathy (VICC) and thrombocytopenia (Maduwage and Isbister, 2014; Warrell, 2010).The saw-scaled viper (*Echis carinatus*) is an additional important cause of haemotoxic snakebite in northern and western India, though it is not endemic to the present study region.

Haemostatic envenomation (HE) is the major determinant of antivenom requirement and early morbidity in snakebite (Isbister, 2010; World Health Organization, 2016). Timely identification is essential, but challenging. The 20-min whole-blood clotting test (20WBCT) and modified Lee–White clotting time (MLW) used in South Asian emergency practice are specific for established coagulopathy but lack sensitivity for early or mild haemostatic activation (Suseel et al., 2023). Standard laboratory coagulation tests — prothrombin time (PT), INR, activated partial thromboplastin time (aPTT), fibrinogen, platelet count — are informative but require processing infrastructure and time that may be unavailable in first-contact healthcare settings (Maduwage and Isbister, 2014).

D-dimer is a stable cross-linked fibrin degradation product generated when plasmin cleaves cross-linked fibrin (Isbister, 2010). Venom metalloproteinases and serine proteases activate the coagulation cascade and cleave fibrinogen, generating fibrin that is subsequently degraded by plasmin to produce D-dimer. This sequence predicts that D-dimer should rise early in haemostatic envenomation, potentially before conventional clotting parameters have deteriorated below conventional diagnostic thresholds (Maduwage and Isbister, 2014).

The Australian Snakebite Project has reported an optimal D-dimer threshold of approximately 2500 ng/mL for VICC detection (Isbister et al., 2022). The present study reports the diagnostic accuracy of admission D-dimer to detect haemostatic envenomation in a prospective Indian emergency department cohort, with comparison against all standard coagulation and haematological tests.

The primary aim was to evaluate the diagnostic accuracy of admission POC D-dimer for identifying haemostatic envenomation (HE). Secondary aims were to compare its performance with standard coagulation tests and to evaluate it against the broader clinically significant envenomation (CSE) endpoint as a pre-specified secondary analysis.

## 2 Methods

### 2.1 Study design and setting

This prospective diagnostic accuracy study was conducted at the Emergency Department of Jubilee Mission Medical College & Research Institute (JMMC&RI), Thrissur, Kerala, India — a tertiary-care teaching hospital with high snakebite endemicity — from March to December 2022. The study was conducted under the ICMR funded Haemostatic Variables in Snakebite Study (HISS) programme (Project No. 5/4/8-21/2021-NCD-11) and was approved by the Institutional Ethics Committee (IEC No. 01/20/IEC/JMMC&RI). Written informed consent was obtained from all participants. Reporting follows the Standards for Reporting of Diagnostic Accuracy Studies (STARD) 2015 guidelines (Bossuyt et al., 2015); a completed STARD checklist is provided as Supplementary File 1.

### 2.2 Participants

Consecutive patients presenting within 6 h of a history suggestive of snakebite were screened. Patients were included if they had presented within 6 h of the bite, and had provided written informed consent, parental consent, and/or assent as applicable. Patients were excluded if the exposure involved bites or stings other than snakebite; if antivenom or blood products had been administered at another institution before sample collection; or if they had a known haematological malignancy or coagulation disorder, chronic liver disease, chronic renal failure, or current use of warfarin, heparin, direct oral anticoagulants, or other injectable anticoagulants or thrombotic illness within three months. Snake species were identified, where possible, using patient-brought specimens/clear photographs reviewed by a trained herpetologist or zoologist. The specific method of species identification (specimen or photograph) was not recorded per patient and cannot be reported retrospectively.

### 2.3 Index test

Venous blood was collected within 30 min of ED arrival. D-dimer was measured, at the point-of-care laboratory within the Emergency Department by nursing staff or junior doctors as part of the HISS research protocol, using the Quidel® Triage D-dimer Test, a single-use fluorescence immunoassay device for quantifying D-dimer in ethylenediamine tetraacetic acid (EDTA)-anticoagulated whole blood, with results displayed in approximately 20 min over a measurable range of 100–5000 ng/mL. The manufacturer's reference interval for the Quidel® Triage D-dimer assay is < 500 ng/mL Fibrinogen Equivalent Units (FEU), above which values are considered elevated in the context of thromboembolic disease. The study-specific threshold of 620 ng/mL was identified by ROC analysis and lies above this reference interval. EDTA-anticoagulated whole blood was used exclusively as the test matrix; plasma was not tested in this study.

D-dimer is not part of the standard hospital snakebite management protocol; all clinical decisions — including antivenom administration — were made in accordance with the institutional standard protocol based on MLW/20WBCT, clinical assessment, and other standard coagulation tests. Standard coagulation tests — PT, INR, aPTT, fibrinogen (Clauss method) performed on ACL TOP 350- Werfen USA with manufacturer supplied reagents like SynthASil, Fibrinogen- C and Recombiplastin, and platelet count(measured using HORIBA MEdical, Japan) — were measured from the same admission sample.

### 2.4 Operational definitions

#### 2.4.1 Hemostatic envenomation (HE)

HE was defined as the presence of any one or more of the following pre-specified, guideline-informed criteria for the duration of index hospital admission.

(a) MLW ≥15 min at admission or six-hourly reassessment (Suseel et al., 2023);-fresh venous blood in three glass tubes, tilted at 1-min intervals; clotting time ≥15 min considered abnormal
(b) Failed 20WBCT (non-clotting blood at 20 min) at admission or six-hourly reassessment;*(*1 mL *fresh venous blood in a clean dry glass tube; failure to clot at 20 min considered positive)*
(c) INR ≥1.4 on the admission sample;
(d) Fibrinogen <100 mg/dL (<1 g/L) on the admission sample;
(e) Platelet count <100 × 10^9^/L (<1 lakh/μL) on the admission sample, consistent with clinically significant thrombocytopenia (Berling and Isbister, 2015);
(f) Bleeding manifestations (haematuria, haemoptysis, gingival bleeding, or epistaxis attributable to envenomation)(World Health Organization, 2016).

Bleeding manifestations were considered HE-related only when adjudicated by the clinician as temporally and clinically consistent with haemotoxic envenomation and not attributable to local trauma or alternative diagnoses.

#### 2.4.2 Clinically significant envenomation (CSE)

As a pre-specified secondary analysis per the ICMR Haemostatic Variables in Snakebite Study (HISS) protocol (Project No. 5/4/8-21/2021-NCD-11), D-dimer performance was also evaluated against the clinically significant envenomation (CSE) composite endpoint. This definition is consistent with WHO criteria for snakebite envenomation outcomes (World Health Organization, 2016) and with the global core outcome framework for snakebite research, and has been applied in prior analyses from the ICMR-HISS programme.

A patient was classified as CSE-positive if they developed any clinically important venom-related outcome during the index admission, including: (i) **Haemostatic abnormality** — spontaneous systemic bleeding (haematuria, gingival bleeding, haemoptysis, or epistaxis attributable to envenomation), non-clotting 20WBCT, prolonged modified Lee-White clotting time, INR >1.4, thrombocytopenia, or hypofibrinogenaemia; (ii) **Neurotoxic envenomation** — bilateral ptosis, external ophthalmoplegia, descending paralysis, or respiratory muscle weakness requiring ventilatory support; (iii) **Cardiovascular abnormality** — hypotension, haemodynamic shock, cardiac arrhythmia, or electrocardiographic abnormalities attributable to envenomation; (iv) **Acute kidney injury** — oliguria or anuria, or rising serum creatinine attributable to envenomation; (v) **Haemoglobinuria or myoglobinuria** — dark urine with positive dipstick findings, or laboratory evidence of intravascular haemolysis or rhabdomyolysis; (vi) **Clinically significant local envenomation** — progressive limb swelling involving more than half the bitten limb within 48 h, swelling following digital bites, rapid extension of swelling beyond the wrist or ankle within hours, or tender regional lymphadenopathy draining the bitten limb.

Unlike the primary HE endpoint, CSE encompasses non-haemostatic venom syndromes; D-dimer's biological relationship with these components is indirect, and findings from the CSE analysis should be interpreted accordingly. As HE criteria constitute a subset of the CSE composite, all patients meeting the HE reference standard are by definition CSE-positive; the converse does not apply.

### 2.5 Statistical analysis

Receiver operating characteristic (ROC) analysis was performed for D-dimer and each comparator against the binary HE outcome. AUCs were estimated with 95% confidence intervals (CI) by the Hanley–McNeil method (Hanley and McNeil, 1982). The Youden index (J = sensitivity + specificity − 1) identified the optimal operating threshold. Sensitivity, specificity, PPV, NPV, likelihood ratios (LR+, LR−), and accuracy at Youden-optimal thresholds, all with 95% Wilson score CIs for proportions, are reported. Spearman rank correlation (r_s_) quantified associations between D-dimer and each comparator.Multivariable binary logistic regression was performed to identify independent predictors of HE. Youden-optimal thresholds identified from ROC analysis of the study data were used to dichotomise continuous predictors as binary variables; as no thresholds were pre-specified before data collection, all values are data-derived and are reported in the Results. Given the data-derived thresholds and limited events-per-variable ratio (n = 11), the model is strictly exploratory and hypothesis-generating. Formal pairwise AUC comparison testing was not performed because the study was exploratory and not powered for comparative ROC inference. A pre-specified sensitivity analysis excluding all non-venomous species (sand boa, wolf snake, Kerala mud snake, bronze back tree snake, rat snake, checkered keelback) assessed robustness. Between-group comparisons used the Mann–Whitney *U* test (continuous) and Fisher's exact test (categorical). All analyses were conducted in Python 3.11 (scikit-learn, scipy) (Pölsterl, 2020),. Statistical significance: p < 0.05 (two-tailed).

Sample size was evaluated post hoc. With 33 HE-positive patients and an observed AUC of 0.874, the Hanley–McNeil 95% CI has a half-width of 0.083, which is considered acceptable precision for an exploratory pilot study. Continuous variables are presented as mean ± SD or median [IQR]; categorical variables as n (%). PT, INR, and aPTT distributions are not described as HE component summaries in Section 3.2: INR is an HE criterion, making its distribution in the HE group subject to incorporation bias; and nine HE patients had PT, INR, and aPTT at the analyser upper detection limit (PT = 180s, INR = 18, aPTT = 180s — censored values), precluding meaningful distributional summary given the resulting bimodal distribution.

## 3 Results

### 3.1 Study cohort

Between March and December 2022, ninety-six consecutive patients met eligibility criteria and were enrolled. Patient flow is shown in Fig. 1. Thirty-three patients (34.4%) met the HE reference standard; 63 (65.6%) did not. D-dimer was available for 95 patients; one non-HE patient was excluded due to insufficient sample volume (D-dimer available for 33 HE and 62 non-HE patients). Baseline demographic and clinical characteristics are presented in Table 1. Median time from bite to ED arrival was 45 [IQR 30–70] minutes. Snake species were identified in 57 patients (59.4%). Among identified species, 11/15 Russell's viper patients (73.3%) and 6/12 HNPV patients (50.0%) met HE criteria; no cobra, krait, Malabar pit viper, or non-venomous bite met HE criteria, validating the biological specificity of the HE reference standard. Antivenom was administered to 28/33 HE patients (84.8%); five HNPV patients did not receive antivenom given limited Indian polyvalent efficacy against Hypnale venom. Among the 63 non-HE patients, 11 (17.5%) received antivenom on clinical grounds; all 11 met CSE criteria.

**Fig. 1:**
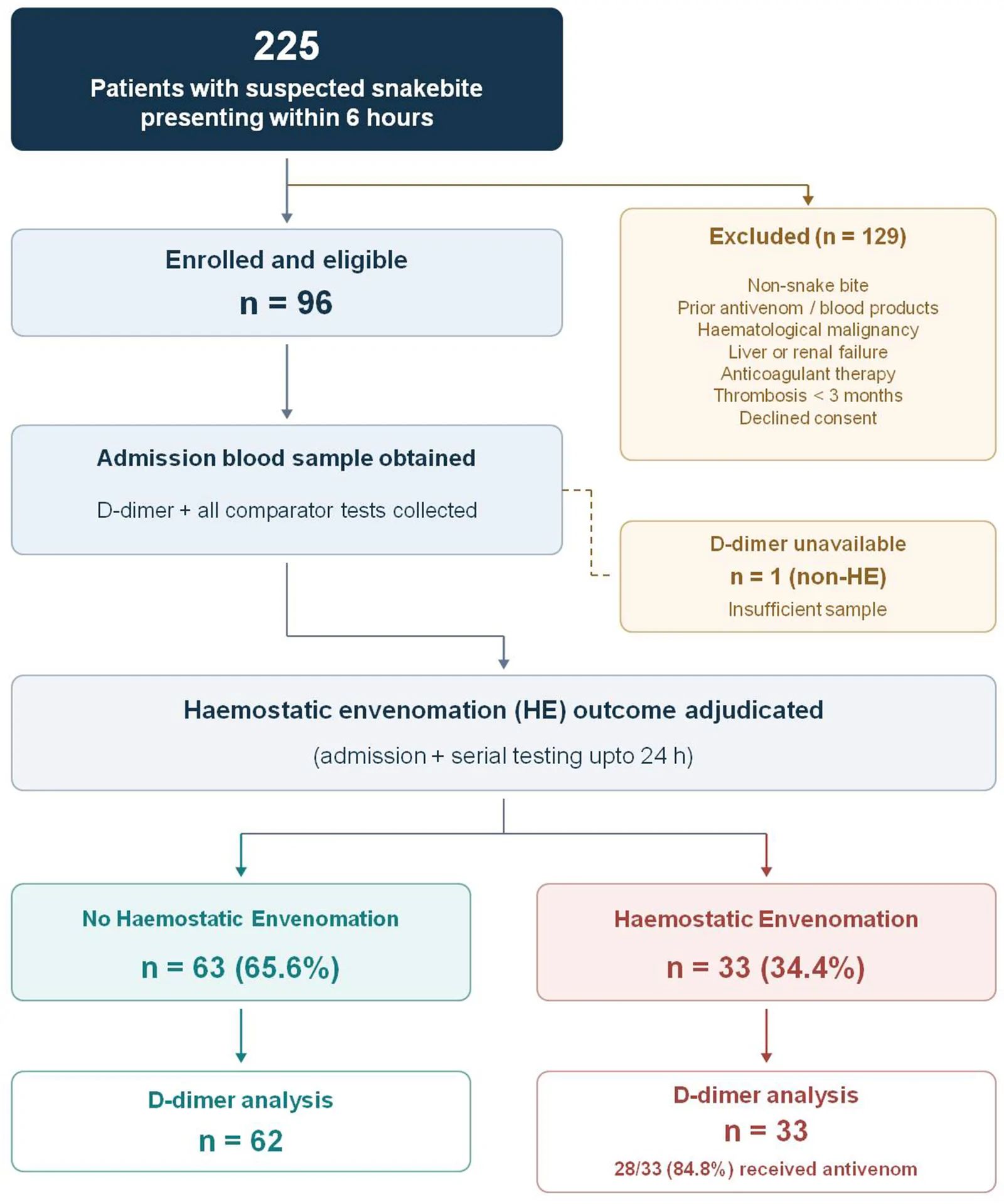
Patient flow diagram (STARD). HE = haemostatic envenomation; MLW = modified Lee–White clotting time; 20WBCT = 20-min whole-blood clotting test.

**Table 1:** Baseline characteristics of enrolled patients by haemostatic envenomation (HE) status.

Characteristic	HE (n = 33)	Non-HE (n = 63)	p-value
Age (years), mean ± SD	46.0 ± 20.6	41.0 ± 16.5	0.195
Male sex, n (%)	21 (63.6)	42 (66.7)	0.823
Time to ED (min), median [IQR]	57 [32–76]	43 [30–68]	0.228
Snake identified, n (%)	17 (51.5)	40 (63.5)	0.281
*Species breakdown, n (% of group)*			
Russell's Viper (*D. russellii*)	11 (33.3)	4 (6.3)	<0.001
HNPV (*H. hypnale*)	6 (18.2)	6 (9.5)	0.329
Common Cobra (*N. naja*)	0 (0.0)	3 (4.8)	0.549
Common Krait (*B. caeruleus*)	0 (0.0)	3 (4.8)	0.549
Malabar Pit Viper	0 (0.0)	1 (1.6)	>0.999
Non-venomous species^a^	0 (0.0)	23 (36.5)	<0.001
Snake unidentified	16 (48.5)	23 (36.5)	0.281
Antivenom administered, n (%)	28 (84.8)	11 (17.5)	<0.001
Local tissue damage, n (%)	20 (60.6)	14 (22.2)	<0.001

Data are mean ± SD, median [IQR], or n (%) as appropriate. Independent-samples *t*-test for age; Mann–Whitney *U* test for time to ED; Fisher's exact test for all categorical variables.

Percentages for species rows are calculated as a proportion of all patients in each group (HE n = 33; non-HE n = 63), not of identified bites only.

HE = haemostatic envenomation; HNPV = hump-nosed pit viper; ED = emergency department; IQR = interquartile range.

a Non-venomous species: sand boa (*Eryx spp.*, n = 11), Kerala mud snake (n = 4), common wolf snake (*Lycodon spp*., n = 4), checkered keelback (*Fowlea piscator*, n = 2), bronze back tree snake (*Dendrelaphis tristis*, n = 1), rat snake (*Ptyas mucosus*, n = 1).

### 3.2 Haemostatic envenomation: components and clinical course

Among the 33 HE patients, the following criteria were met at admission or 6-h reassessment (criteria not mutually exclusive): MLW ≥15 min in 16 (48.5%), fibrinogen <100 mg/dL in 12 (36.4%), failed 20WBCT in 10 (30.3%), INR ≥1.4 in 10 (30.3%), and platelet <100 × 10^9^/L in 3 (9.1%). Admission laboratory parameters by HE status are detailed in Table 2.

**Table 2:** Admission laboratory parameters by haemostatic envenomation (HE) status.

Laboratory Parameter	HE (n = 33)	Non-HE (n = 63)	p-value
D-dimer (ng/mL)^a^	1980 [1340–2810]	100 [100–467]	<0.001
MLW (min)	14 [13–35]	13 [12–13]	<0.001
Failed 20WBCT, n (%)	10 (30.3)	0 (0.0)	<0.001
aPTT (s)^b^	30 [25–180]	34 [31–36]	0.487
Fibrinogen (mg/dL)	180 [30–255]	256 [216–304]	<0.001
Platelet count (×10^9^/L)	196 [158–259]	243 [207–281]	0.004
PT (s)^c^	12 [11–180]	12 [11–12]	0.065
INR§	1 [1–18]	1 [1–1]	0.019

Data are median [IQR] unless otherwise stated. Mann–Whitney *U* test for continuous variables; Fisher's exact test for categorical variables.

The wide IQR for PT and INR in the HE group reflects a bimodal distribution: 23/33 patients had values within the normal reference range (PT 11–13 s, INR 0.9–1.3), while nine of the most severely envenomated patients had PT and INR at the analyser upper detection limit (180 s and 18 respectively) due to complete fibrinogen depletion. Median values should be interpreted accordingly.

a D-dimer: n = 95 (one non HE patient excluded: insufficient sample volume).

b aPTT available for all 96 patients; nine HE patients at upper detection limit (180 s).

c Nine HE patients had PT = 180 s and INR = 18 (upper detection limit of the coagulation analyser; blood severely defibrinated due to VICC). PT and INR available for all 96 patients. PT and INR distributions are not summarised in Section 3.2 due to incorporation bias (INR is an HE criterion) and the bimodal distribution produced by nine patients at the analyser upper detection limit (PT = 180 s, INR = 18, aPTT = 180 s — censored values indicating severely defibrinated blood) MLW = modified Lee–White clotting time; 20WBCT = 20-min whole-blood clotting test; aPTT = activated partial thromboplastin time; PT = prothrombin time; INR = international normalised ratio.

Four patients had a normal admission MLW and 20WBCT but developed delayed coagulopathy at the 6-h reassessment (two HNPV, two Russell's viper). All four were classified HE (delayed criterion). Of these, three (75.0%) had admission D-dimer ≥620 ng/mL (1,923, 2,670, and 1030 ng/mL respectively); one had D-dimer of 447 ng/mL.

### 3.3 D-dimer distributions and correlations

Admission D-dimer was substantially higher in HE patients: median 1980 [IQR 1340–2810] ng/mL versus 100 [IQR 100–467] ng/mL in non-HE patients (p < 0.001; Table 2). D-dimer showed significant positive correlation with MLW (r_s_ = +0.363, p < 0.001) and negative correlation with platelet count (r_s_ = −0.265, p = 0.009). Correlations with PT, INR, fibrinogen, and aPTT were not statistically significant. D-dimer concentrations by HE status are illustrated in Fig. 3.

### 3.4 Diagnostic performance: D-dimer and comparators

At the Youden-optimal threshold of 620 ng/mL (n = 95), D-dimer correctly classified 78 of 95 patients: 29 true positives, 4 false negatives, 49 true negatives, and 13 false positives. ROC curves are illustrated in Fig. 2; Youden-optimal thresholds and diagnostic accuracy metrics for each comparator are summarised in Table 3. D-dimer achieved an AUC of 0.874 (95% CI: 0.791, 0.957), sensitivity 87.9% (95% CI: 72.7%, 95.2%), specificity 79.0% (95% CI: 67.4%, 87.3%), and NPV 92.5% (95% CI: 82.1%, 97.0%) — the highest among all comparators. The NPV is the primary clinically actionable finding: a D-dimer below 620 ng/mL reduces the post-test probability of HE from 34.4% to approximately 8%. D-dimer outperformed all comparators: Lee-White clotting time (MLW, AUC 0.763), 20WBCT (0.652), fibrinogen (0.728), platelet count (0.681), INR (0.646), and PT (0.615). Full performance metrics are in Table 3.

**Fig. 2:**
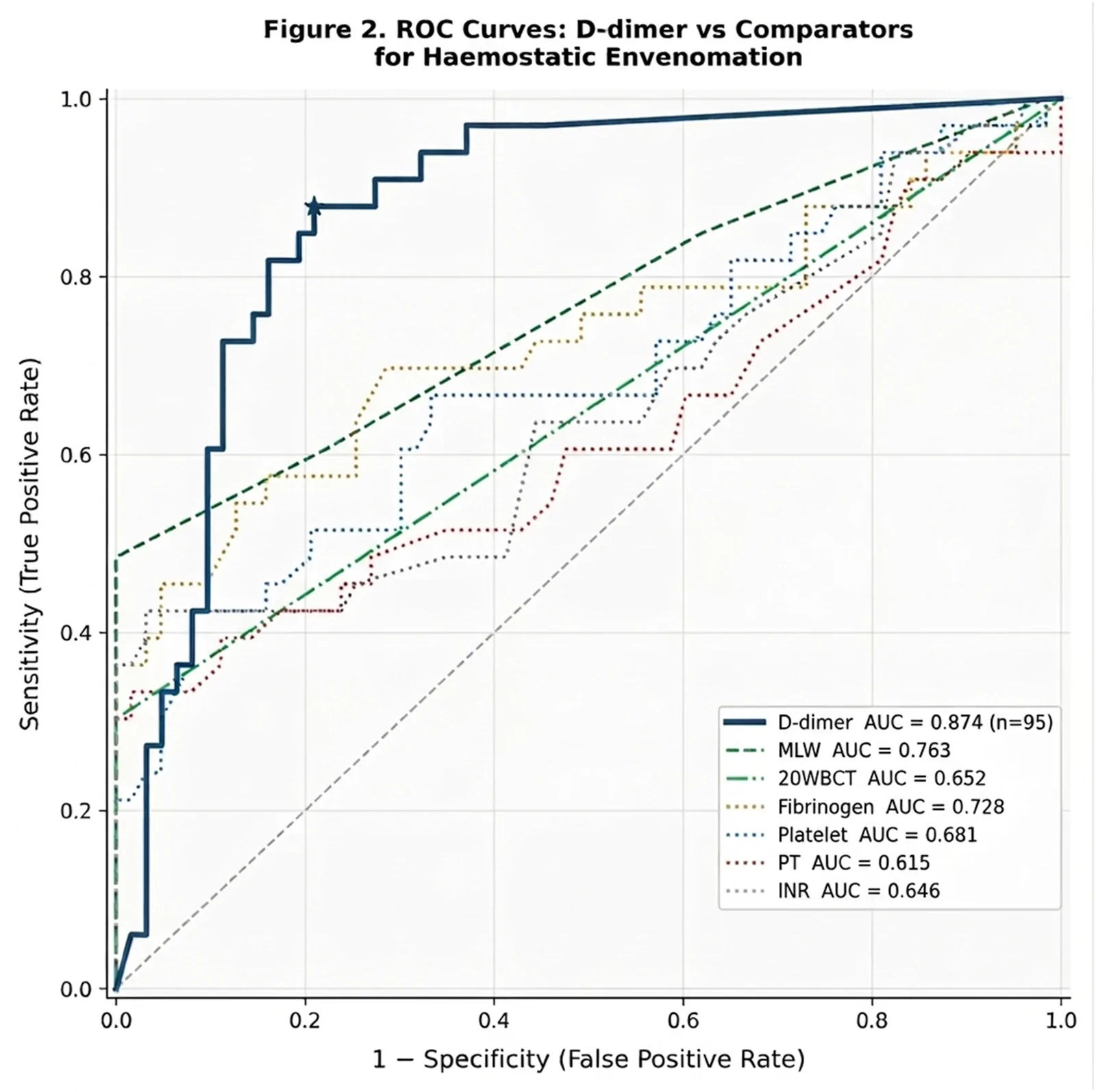
Receiver operating characteristic (ROC) curves for D-dimer and comparator tests against the haemostatic envenomation (HE) reference standard. The diagonal dashed line represents chance discrimination (AUC = 0.50). MLW (Modified Lee-White clotting time) and 20WBCT (20-min whole-blood clotting test) are presented as separate curves. aPTT is not shown due to its bidirectional relationship with HE severity (AUC 0.544; see Table 3). ★ Youden-optimal threshold for D-dimer (620 ng/mL). Sample sizes: n = 95 for D-dimer; n = 96 for all others. AUC = area under the ROC curve; CI = 95% confidence interval.

**Fig. 3:**
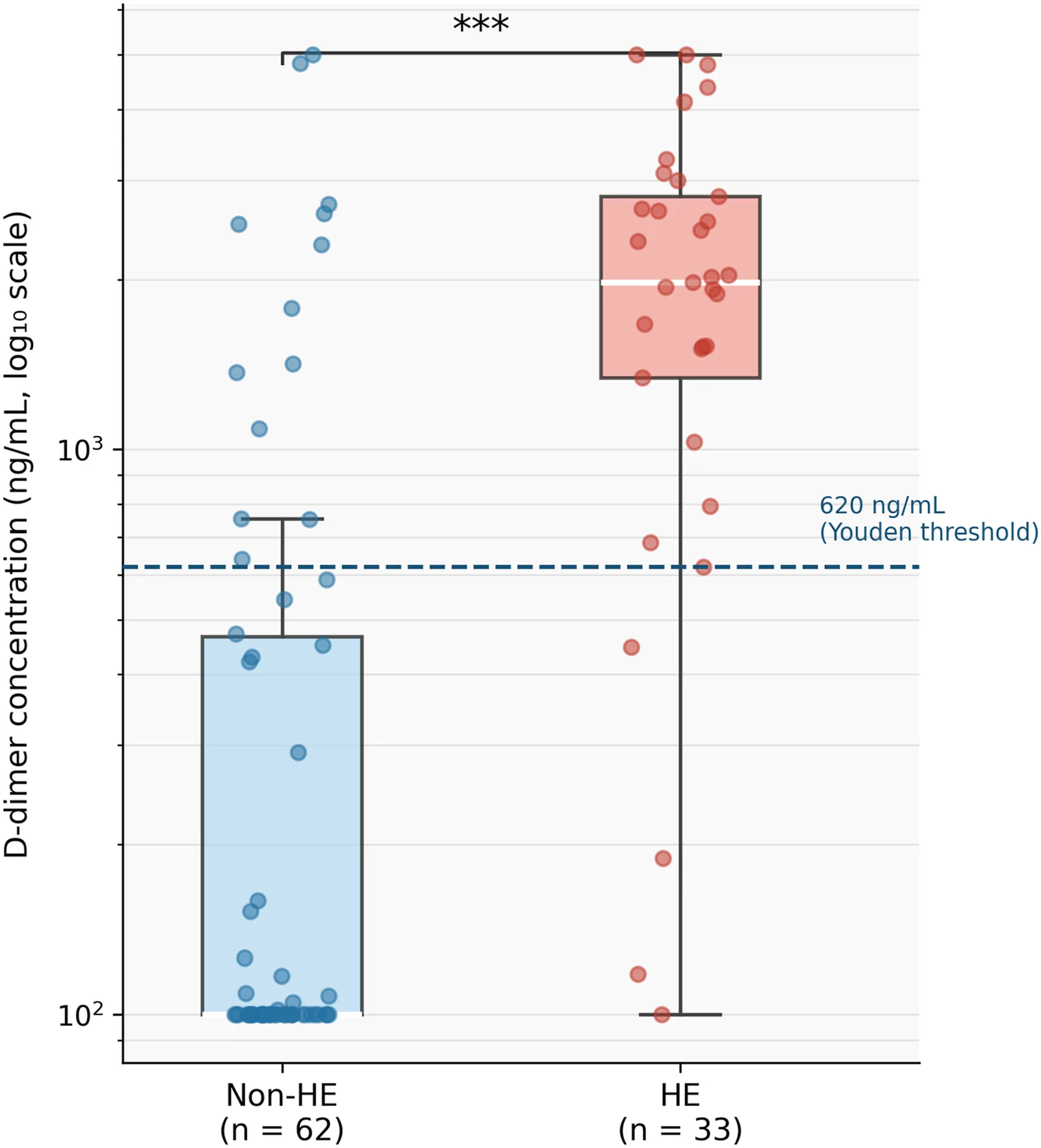
Admission D-dimer concentrations (ng/mL, log_10_ scale) by haemostatic envenomation (HE) status (n = 95). Boxes display median and interquartile range; whiskers extend to 1.5 × IQR; individual data points are superimposed. The horizontal dashed line indicates the Youden-optimal threshold of 620 ng/mL ∗∗∗ p < 0.001 (Mann–Whitney *U* test). HE = haemostatic envenomation; IQR = interquartile range.

**Table 3:** Diagnostic accuracy of D-dimer and comparator tests for haemostatic envenomation (n = 96 unless stated).

Test	AUC (95% CI)	Optimal Cut-off	Sensitivity % (95% CI)	Specificity % (95% CI)	PPV % (95% CI)	NPV % (95% CI)	J
D-dimer (n = 95)^a^	0.874 (0.791–0.957)	≥620 ng/mL	87.9 (72.7–95.2)	79.0 (67.4–87.3)	69.0 (54.0–80.9)	92.5 (82.1–97.0)	0.669
MLW (n = 96)^b^	0.763 (0.657–0.870)	≥15 min	48.5 (32.5–64.8)	100.0 (94.3–100.0)	100.0 (80.6–100.0)	78.8 (68.6–86.3)	0.485
20WBCT — failed (n = 96)^b^	0.652 (0.533–0.770)	Failed	30.3 (17.4–47.3)	100.0 (94.3–100.0)	100.0 (72.2–100.0)	73.3 (63.1–81.5)	0.303
aPTT (n = 96)^c^	0.544 (0.421–0.666)	≤30.5 s	42.4 (27.2–59.2)	84.1 (73.2–91.1)	58.3 (38.8–75.5)	73.6 (62.4–82.4)	0.266
Fibrinogen (n = 96)	0.728 (0.616–0.839)	≤186 mg/dL	54.5 (38.0–70.2)	87.3 (76.9–93.4)	69.2 (50.0–83.5)	78.6 (67.6–86.6)	0.418
Platelet count (n = 96)	0.681 (0.564–0.797)	≤176 × 10^9^/L	42.4 (27.2–59.2)	92.1 (82.7–96.6)	73.7 (51.2–88.2)	75.3 (64.6–83.6)	0.345
PT (n = 96)	0.615 (0.494–0.736)	≥15.9 s	33.3 (19.8–50.4)	98.4 (91.5–99.7)	91.7 (64.6–98.5)	73.8 (63.5–82.0)	0.317
INR (n = 96)	0.646 (0.527–0.765)	≥1.2	42.4 (27.2–59.2)	96.8 (89.1–99.1)	87.5 (64.0–96.5)	76.2 (65.9–84.2)	0.392

All cut-offs are Youden-optimal (maximising sensitivity + specificity − 1). All 95% CIs: Wilson score method for proportions; Hanley–McNeil method for AUC.

a D-dimer: n = 95 (one non-HE patient excluded due to insufficient sample volume; D-dimer available for 33 HE and 62 non-HE patients).

b MLW and 20WBCT are presented as separate rows because they assess distinct aspects of clotting. MLW (Modified Lee-White clotting time) is a quantitative continuous test measuring time to clot formation; 20WBCT is a binary qualitative test for complete failure of clot formation at 20 min. The 100.0% specificity for both MLW and 20WBCT reflects partial incorporation of these criteria into the HE reference standard and should be interpreted with caution (MLW ≥15 min and failed 20WBCT are both HE criteria). For MLW, clot lysis cases were assigned a value of 35 min for continuous ROC analysis; all such cases also met the failed 20WBCT criterion.

c aPTT demonstrated bidirectional behaviour in this cohort: shortening (≤30.5 s) in early/moderate VICC due to early coagulation factor activation, and reaching the analyser upper detection limit (180 s) in the nine most severely envenomated patients with complete fibrinogen depletion. The AUC of 0.544 reflects near-chance discrimination, consistent with this U-shaped distribution across HE severity. aPTT is not recommended as a standalone discriminator for HE. AUC, area under the ROC curve; CI, 95% confidence interval; D-dimer values in ng/mL FEU; fibrinogen in mg/dL; HE, haemostatic envenomation; INR, international normalised ratio; J, Youden index; MLW, modified Lee-White clotting time; NPV, negative predictive value; PPV, positive predictive value; PT, prothrombin time; ROC, receiver operating characteristic; 20WBCT, 20-min whole-blood clotting test; aPTT, activated partial thromboplastin time; VICC, venom-induced consumption coagulopathy.

MLW at ≥15 min achieved 100.0% specificity but only 48.5% sensitivity, confirming its value as a rule-in but not rule-out test. The 20WBCT (failed) achieved 100.0% specificity and 30.3% sensitivity. The 100.0% specificity for both reflects partial incorporation into the HE reference standard.

Of the 13 false-positive patients, nine were CSE-positive (six received antivenom — haemotoxic species with borderline clotting times and markedly elevated D-dimer, consistent with subclinical haemostatic activation below HE thresholds; three were HNPV patients for whom antivenom was withheld given limited Indian polyvalent efficacy). Four were CSE-negative with no antivenom; two were confirmed non-venomous species (bronze back tree snake, Kerala mud snake) with D-dimer elevation attributable to local wound trauma rather than venom effect. D-dimer is not a venom-specific marker — elevation in non-venomous bites accounts for its 79.0% specificity.

All four false-negative patients had haemotoxic bleeding as their primary HE criterion with near-normal or undetectable D-dimer, reflecting either localised haemotoxic effects without sufficient systemic fibrinolysis, or a non-fibrinolytic mechanism (direct fibrinogenolysis or TMA) in one patient with paradoxically low D-dimer despite fibrinogen depletion.

### 3.5 D-dimer in diagnosing haemostatic envenomation

As a supplementary exploratory analysis (n = 95 complete cases), D-dimer ≥620 ng/mL was the strongest independent predictor of HE (adjusted OR 22.04, 95% CI: 6.13, 79.25; p < 0.001; Table 4). MLW ≥13.5 min was also independently significant (adjusted OR 4.41, 95% CI: 1.30, 14.98; p = 0.018), suggesting that D-dimer and MLW provide partially complementary diagnostic information. Platelet count ≤226.5 × 10^9^/L was not independently significant (adjusted OR 1.91, 95% CI: 0.59, 6.23; p = 0.283).Given that all predictor thresholds were data-derived from the same dataset, the model is subject to internal optimisation and should be considered hypothesis-generating only.

**Table 4:** Multivariable logistic regression: independent predictors of haemostatic envenomation (n = 95).

Variable	β	SE	Wald χ^2^	p-value	Adjusted OR (95% CI)
**D-dimer ≥620 ng/mL**	3.093	0.653	22.44	<0.001	22.04 (6.13–79.25)
MLW ≥13.5 min	1.483	0.624	5.65	0.018	4.41 (1.30–14.98)
Platelet ≤226.5 × 10^9^/L	0.648	0.603	1.15	0.283	1.91 (0.59–6.23)
Constant	−3.286	0.657	24.97	<0.001	0.04 (0.01–0.14)

n = 95 (complete cases for D-dimer, MLW, and platelet count). Logistic regression with maximum-likelihood estimation; 95% CIs for OR derived from Wald standard errors.

INR, fibrinogen, and platelet at their HE-criterion thresholds were excluded from the model to avoid predictor–outcome circularity. aPTT was excluded as it is not a component of the HE reference standard in this study.

OR = odds ratio; CI = confidence interval; SE = standard error; MLW = modified Lee–White clotting time; HE = haemostatic envenomation.

With 33 HE-positive events across three predictor variables, the events-per-variable ratio (11) is at the lower boundary of acceptable for stable logistic regression estimation. The wide confidence interval for D-dimer (6.13–79.25) reflects this statistical uncertainty. Results should be considered hypothesis-generating and require validation in larger cohorts.

### 3.6 Sensitivity analysis

In the pre-specified sensitivity analysis excluding 23 confirmed non-venomous species (n = 73; 33 HE, 40 non-HE; D-dimer available n = 72), D-dimer AUC was 0.837 (95% CI: 0.740–0.933) with sensitivity 87.9% and specificity 71.8% at 620 ng/mL, consistent with the primary analysis.

### 3.7 Secondary analysis: D-dimer performance against the composite clinically significant envenomation endpoint

As a pre-specified secondary analysis per the ICMR-HISS protocol, D-dimer performance was evaluated against the CSE endpoint (n = 52, 54.2%). To determine whether D-dimer has a genuine signal beyond the HE subset, a subgroup analysis among the 63 non-HE patients compared 19 who met CSE criteria with 44 who did not. D-dimer was significantly higher in non-HE CSE patients (median 590 [156–1800] ng/mL) than non-CSE patients (100 [100–108] ng/mL; p < 0.001), with AUC 0.798 (95% CI: 0.667–0.929). These patients had entirely normal conventional coagulation parameters; their elevated D-dimer represents subclinical haemostatic activation below HE thresholds, confined to haemotoxic species. Purely neurotoxic patients had D-dimer at the assay floor. At a Youden-optimal threshold of 152 ng/mL in the non-HE subgroup, sensitivity was 78.9% and specificity 83.7%.

## 4 Discussion

Admission point-of-care D-dimer achieved an AUC of 0.874 for identifying haemostatic envenomation (HE), with numerically higher discriminatory performance than all standard coagulation comparators in this prospective Indian emergency department cohort. The most clinically actionable finding was the NPV of 92.5% at the 620 ng/mL threshold: a negative D-dimer reduces the post-test probability of HE from 34.4% to approximately 8%, supporting potential utility as a rapid rule-out biomarker at ED presentation. Importantly, this association persisted after adjustment for standard coagulation measures, suggesting that D-dimer may capture aspects of early haemostatic disturbance not fully reflected by conventional tests especially in this early presenting cohort.

The HE composite, defined prospectively and informed by WHO haemotoxic envenomation criteria (World Health Organization, 2016) and published VICC definitions (Maduwage and Isbister, 2014), is broader than the VICC construct used in the Australian Snakebite Project, additionally incorporating thrombocytopenia and haemotoxic bleeding as independent endpoints. The species distribution validates the definition biologically — all non-venomous animals and neurotoxic species were correctly classified as non-HE, while Russell's viper (73.3%) and HNPV (50.0%) carried the HE burden as expected.

The Youden-optimal threshold of 620 ng/mL is substantially higher than the assay detection floor (100 ng/mL) and is consistent with published snakebite literature. Isbister et al. reported an optimal D-dimer threshold of 2500 ng/mL for VICC in the Australian Snakebite Project (Isbister et al., 2022), with sensitivity and specificity exceeding 90% in a predominantly elapid cohort. The lower threshold in the present study plausibly reflects the heterogeneous venom mix of Indian species and the stricter HE reference standard, which correctly classifies patients with early or subclinical activation as non-HE. Patients in the present study presented within 6 h of the bite, potentially before D-dimer had peaked; the Quidel Triage fluorescence immunoassay may have different FEU calibration characteristics compared with the assay used in ASP-29. Thirteen non-HE patients had D-dimer ≥620 ng/mL; several were bitten by Russell's viper or HNPV with D-dimer values up to 5000 ng/mL despite not meeting HE criteria at admission. These cases likely represent early haemostatic activation detected by D-dimer before conventional coagulation parameters cross guideline thresholds, a phenomenon described previously in the VICC literature and consistent with D-dimer's mechanistic coupling to early fibrin turnover (Isbister et al., 2013; Little, 2022).

MLW (Lee-White clotting time) at the ≥15-min threshold showed limited sensitivity despite high specificity, as did the failed 20WBCT, indicating that both tests are better suited to identifying established haemostatic disturbance than excluding HE at admission. The independent significance of MLW ≥13.5 min in multivariable analysis suggests that D-dimer and MLW capture partially complementary diagnostic information. The complementary profiles of D-dimer (high sensitivity, moderate specificity) and both bedside clotting tests (high specificity, limited sensitivity) support investigation of a sequential triage approach — D-dimer for early risk stratification followed by MLW or 20WBCT for confirmatory rule-in — though prospective evaluation of this strategy is required before clinical recommendations can be made. The PT/INR has high specificity but limited sensitivity due to censored values at the extreme end. The aPTT (AUC 0.544) showed near-chance discrimination due to its bidirectional behaviour in VICC; shortening in early factor consumption and reaching the analyser upper detection limit (180 s) in patients with complete fibrinogen depletion confirming it as an unreliable standalone discriminator for HE.

The NPV of 92.5% is the most directly actionable clinical finding. In this cohort, a D-dimer below 620 ng/mL reduces the post-test probability of HE from 34% to 8%, supporting its potential role as an early rule-out test, pending prospective validation. Conversely, the LR+ of 4.19 indicates that a positive D-dimer provides meaningful, though not conclusive, evidence for HE risk. The four patients who developed delayed coagulopathy (two HNPV, two RVV) had entirely normal admission MLW and 20WBCT. Three already had D-dimer ≥620 ng/mL at admission (1923; 2670; and 1030 ng/mL) while conventional tests were still normal. D-dimer rises early because it reflects fibrin generation and degradation — initiated by venom thrombin activation — before sufficient clotting factor consumption has prolonged bedside clotting tests (Thonon et al., 2025). The one patient with D-dimer 447 ng/mL may represent haemostatic activation beginning after the admission sample or activation insufficient to generate substantial systemic fibrinolysis.

Serial D-dimer measurement — as performed in ASP-29 (Isbister et al., 2022) at admission, 6, and 24 h — may further improve diagnostic yield. In our cohort, serial D-dimer data were collected per the ICMR-HISS protocol but were not analysed here as the study specifically addresses admission triage performance which also has a pragmatic screening application in resource limited settings. Analysis of D-dimer kinetics and post-treatment monitoring is planned as a separate study.

A practical consideration for implementation is the cost of the Quidel® Triage platform (∼INR 1000–1200 per cartridge). Lower-cost quantitative D-dimer assays on automated laboratory platforms may offer an alternative, though their assay-specific thresholds would require independent validation. Prospective health-economic evaluation should weigh test cost against avoidable antivenom administration and unnecessary monitoring.

The secondary analysis against the broader CSE endpoint — which includes non-haemostatic outcomes such as neurotoxic envenomation and local tissue damage alongside coagulopathy — yielded an AUC of 0.885, marginally higher than the primary HE-specific AUC of 0.874. D-dimer demonstrated an AUC of 0.798 among non-HE CSE patients versus non-CSE patients, confirming that the CSE signal is not driven entirely by the HE subset. However, the elevated D-dimer in this subgroup was confined to haemotoxic species (Russell's viper, HNPV) with borderline clotting times, not to neurotoxic presentations. Purely neurotoxic patients had D-dimer at the assay floor, indicating that D-dimer does not reflect general systemic venom activity but rather detects haemostatic activation across a spectrum — from overt HE at the extreme to subclinical fibrin turnover in haemotoxic species before conventional thresholds are crossed. The lower CSE threshold of 139 ng/mL therefore reflects this earlier detection point rather than a qualitatively different mechanism.

### 4.1 Limitations

This study has several limitations. First, the sample is relatively small (n = 96; n = 33 HE-positive); while adequate for an exploratory pilot analysis, the 95% CI for sensitivity (72.7%–95.2%) is wide, and external validation in larger, multicentre Indian cohorts is necessary before any clinical recommendation.

Second, the study is single-centre and reflects the species profile of a tertiary-care institution in Kerala, India; generalisability to settings with different venom epidemiology (e.g., saw-scaled viper, *Echis carinatus*, in northern India) requires confirmation.

Third, partial incorporation bias applies to MLW/20WBCT, fibrinogen, and platelet count as comparator tests, as these simultaneously contribute to HE classification; their AUCs may be inflated relative to a fully adjudicator-defined reference standard, whereas D-dimer's AUC is unaffected.

Fourth, the Youden-optimal threshold of 620 ng/mL was derived in the study population and may not transfer directly across D-dimer assay platforms or clinical settings without independent validation.

Fifth, D-dimer was measured at a single time point; serial measurements may improve diagnostic yield, particularly for delayed coagulopathy.

The LR-based post-test probability estimates assume a pre-test probability consistent with this cohort and should be adjusted for local epidemiology. The multivariable logistic regression model (Table 4) should be interpreted with caution. All predictor thresholds in the regression model — including D-dimer ≥620 ng/mL — were Youden-derived from the same dataset used in the regression. No thresholds were pre-specified. This constitutes internal optimisation and the model should be interpreted as strictly exploratory, with the primary conclusions resting on the threshold-independent ROC analysis.

Formal administrative blinding of D-dimer results from treating clinicians was not implemented; however, D-dimer has no established role in Indian snakebite management guidelines and HE adjudication is based entirely on objective pre-specified criteria, so the risk of performance bias is considered low.

## 5 Conclusions

Admission D-dimer demonstrated numerically higher discriminatory performance for haemostatic envenomation than all standard coagulation comparators in this prospective Indian emergency department cohort, when evaluated against a rigorously defined, D-dimer-independent composite reference standard. Its negative predictive value of 92.5% at a Youden-optimal threshold of 620 ng/mL supports potential utility as a rapid potential early exclusion biomarker for haemostatic envenomation in the snakebite emergency setting. A proposed sequential triage strategy — D-dimer as a sensitive early filter followed by MLW/20WBCT for confirmatory specificity — merits prospective evaluation. These findings are exploratory and require external validation in larger multicentre cohorts before formal clinical recommendations can be formulated. The consistency of D-dimer's discriminatory performance across both the focused haemostatic envenomation endpoint (AUC 0.874) and the broader clinically significant envenomation endpoint (AUC 0.885) further supports its biological plausibility as a general indicator of systemic venom activity.

## CRediT authorship contribution statement

**Deo Mathew:** Writing – review & editing, Writing – original draft, Formal analysis, Data curation, Conceptualization, Visualization. **Siju V. Abraham:** Writing – review & editing, Supervision, Project administration, Funding acquisition, Data curation, Conceptualization, Investigation, Formal analysis, Methodology, Resources. **Sarah Paul:** Investigation, Formal analysis, Data curation. **Aboobacker Mohamed Rafi:** Writing – review & editing, Methodology, Supervision, Project administration. **Appu Suseel:** Writing – review & editing. **C.K. Kassyap:** Writing – review & editing, Project administration.

## Ethics approval

Institutional Ethics Committee, JMMC&RI (IEC No. 01/20/IEC/JMMC&RI)

## Funding

10.13039/501100001411Indian Council of Medical Research- Haemostatic Variables in Snakebite Study (HISS) ICMR, Project No. 5/4/8-21/2021-NCD-II.

## Declaration of competing interest

The authors declare that they have no known competing financial interests or personal relationships that could have appeared to influence the work reported in this paper.

## Data Availability

Data will be made available on request.
