# Supplementary material for: Admission point-of-care D-dimer for identifying haemostatic envenomation after snakebite: A prospective observational study
Source: Toxicon X. 2026 Sep 28;32:100266. doi: 10.1016/j.toxcx.2026.100266 (PMC13673363; doi:10.1016/j.toxcx.2026.100266)
Supplement: Supplementary file 1 [file mmc1.docx]

**Supplementary File 1: STARD 2015 Checklist**

**Manuscript:** *Admission Point-of-Care D-Dimer for Identifying Haemostatic Envenomation After Snakebite: A Prospective Observational Study*

**Journal:** Toxicon X

**Registration:** ClinicalTrials.gov NCT04520282

**Corresponding author:** Dr Siju V Abraham, sijuvabraham@jmmc.ac.in

**Ethics:** IEC No. 01/20/IEC/JMMC&RI

**Funding:** ICMR-HISS, Project No. 5/4/8-21/2021-NCD-11

**STARD reference:** Bossuyt, P.M., Reitsma, J.B., Bruns, D.E., et al., 2015. STARD 2015: an updated list of essential items for reporting diagnostic accuracy studies. Radiology 277, 826–832. https://doi.org/10.1148/radiol.2015151516

| **No.** | **STARD 2015 Essential Item** | **Location / Response** |
| --- | --- | --- |
| **TITLE OR ABSTRACT** | | |
| 1 | Identification as a study of diagnostic accuracy using at least one measure of accuracy (sensitivity, specificity, predictive values, or AUC). | Title explicitly identifies as a diagnostic accuracy study. Abstract reports AUC 0.874, sensitivity 87.9%, specificity 79.0%, PPV 69.0%, NPV 92.5%. |
| **ABSTRACT** | | |
| 2 | Structured summary of study design, methods, results, and conclusions. | Abstract present. Formatted per journal requirements (≤250 words, no subheadings). Includes: study type and setting; HE reference standard criteria; index test (Quidel® Triage D-dimer); threshold (620 ng/mL); AUC, sensitivity, specificity, PPV, NPV with 95% CIs; OR 22.04 (6.13–79.25); CSE secondary result (AUC 0.885). |
| **INTRODUCTION** | | |
| 3 | Scientific and clinical background, including the intended use and clinical role of the index test. | Introduction paragraphs 1–4. Covers global and Indian snakebite burden (Kasturiratne et al., 2008; Suraweera et al., 2020); predominant haemotoxic species in peninsular India (RVV, HNPV); VICC pathophysiology and D-dimer mechanistic coupling; limitations of 20WBCT and MLW for early detection; proposed triage role of POC D-dimer (Isbister et al., 2022). |
| 4 | Study objectives and hypotheses. | Introduction, final paragraph: “The present study reports the diagnostic accuracy of admission D-dimer to detect haemostatic envenomation in a prospective Indian emergency department cohort, with comparison against all standard coagulation and haematological tests.” |
| **METHODS — Study design** | | |
| 5 | Whether data collection was planned before the index test and reference standard were performed (prospective) or after (retrospective). | Prospective. Methods Section 2.1. Data collection, index test measurement, and reference standard adjudication all pre-specified per ICMR-HISS protocol before enrolment began. |
| 6 | Eligibility criteria. | Methods Section 2.2.  Inclusion: (i) definite/probable snakebite confirmed by attending physician; (ii) presentation within 6 hours of bite; (iii) written informed consent / parental consent / assent as applicable.  Exclusion: (i) bites other than snakebite; (ii) antivenom or blood products administered before sample collection; (iii) known haematological malignancy or coagulation disorder; (iv) chronic liver disease; (v) chronic renal failure; (vi) current anticoagulant therapy (warfarin, heparin, DOAC, injectable anticoagulants); (vii) thrombotic illness within three months.  No age restriction — all age groups included. |
| 7 | On what basis potentially eligible participants were identified. | Consecutive presentation to the ED with a history suggestive of snakebite. Methods Section 2.2. |
| 8 | Where and when potentially eligible participants were identified (setting, location, dates). | Emergency Department, Jubilee Mission Medical College & Research Institute (JMMC&RI), Thrissur, Kerala, India — a tertiary-care teaching hospital in a high-endemicity region. March to December 2022. Methods Section 2.1. |
| 9 | Whether participants formed a consecutive, random, or convenience series. | Consecutive series. Methods Section 2.2. |
| **METHODS — Test methods** | | |
| 10a | Index test, in sufficient detail to allow replication. | Methods Section 2.3. Venous blood collected within 30 minutes of ED arrival. D-dimer measured using the Quidel® Triage D-dimer Test — a single-use quantitative fluorescence immunoassay device for EDTA-anticoagulated whole blood or plasma; measurable range 100–5,000 ng/mL FEU; result in approximately 20 minutes. Laboratory personnel blinded to HE outcome adjudication. |
| 10b | Reference standard, in sufficient detail to allow replication. | Methods Section 2.4. HE adjudicated at 24 hours by two independent senior clinicians blinded to D-dimer.  HE = any one of: (a) MLW ≥15 min; (b) failed 20WBCT; (c) INR ≥1.4; (d) fibrinogen <100 mg/dL; (e) platelet <100 ×10⁹/L; (f) haemotoxic bleeding (haematuria, haemoptysis, gingival bleeding, or epistaxis attributable to envenomation).  Explicitly excluded from HE: D-dimer; ISTH overt-DIC score (incorporates D-dimer); aPTT (bidirectional in VICC). |
| 11 | Rationale for choosing the reference standard. | Methods Section 2.4 and Discussion Section 4. No validated single gold standard for HE exists; composite used. D-dimer excluded to avoid incorporation bias. ISTH DIC excluded as it directly scores D-dimer concentration. Each criterion threshold informed by WHO guidelines (World Health Organization, 2016), VICC literature (Isbister, 2010; Berling and Isbister, 2015), and validated local MLW data (Suseel et al., 2023). |
| 12a | Definition of and rationale for test positivity cut-offs of the index test. | Methods Section 2.5. Youden-optimal threshold: 620 ng/mL (J = 0.669), derived by ROC analysis maximising sensitivity + specificity − 1. Corresponds to the point on the ROC curve geometrically farthest from the diagonal chance line. |
| 12b | Definition of and rationale for test positivity cut-offs of the reference standard. | Methods Section 2.4. Each HE criterion:  MLW ≥15 min: validated threshold (Suseel et al., 2023); WHO-recommended.  Failed 20WBCT: WHO primary bedside criterion (World Health Organization, 2016).  INR ≥1.4: pre-specified in ICMR-HISS protocol; conservative extrinsic pathway threshold.  Fibrinogen <100 mg/dL (<1 g/L): WHO severe VICC fibrinogen depletion threshold (World Health Organization, 2016).  Platelet <100 ×10⁹/L: clinically significant thrombocytopenia (Berling and Isbister, 2015).  Haemotoxic bleeding: WHO-defined systemic haemotoxic manifestation (World Health Organization, 2016). |
| 13a | Whether reference standard results were available to the performers/readers of the index test. | Methods Section 2.3. Laboratory personnel performing D-dimer measurement were blinded to HE outcome adjudication. |
| 13b | Whether index test results were available to the assessors of the reference standard. | Methods Sections 2.3–2.4. Treating clinicians adjudicating HE were blinded to D-dimer values at the time of adjudication. |
| **METHODS — Analysis** | | |
| 14 | Methods for estimating or comparing measures of diagnostic accuracy. | Methods Section 2.5. ROC analysis; Hanley–McNeil AUC with 95% CI (Hanley and McNeil, 1982); Youden index for optimal thresholds; Wilson score 95% CIs for proportions; Spearman rank correlation; multivariable logistic regression; Mann–Whitney U; Fisher’s exact test. Python 3.11 (scikit-learn, scipy). |
| 15 | How indeterminate index test or reference standard results were handled. | One patient excluded from D-dimer analysis (insufficient sample volume). No indeterminate HE adjudications. Results Section 3.1. |
| 16 | How missing data were handled. | D-dimer: n=95/96 (one excluded — insufficient sample; explicitly reported).  PT, INR, aPTT: nine HE patients had values at the analyser upper detection limit (PT=180 s, INR=18, aPTT=180 s) due to severely defibrinated blood. Included with recorded values; bimodal distribution documented in Table 2 footnote. No imputation performed. |
| 17 | Analyses of variability in diagnostic accuracy, specifying covariates. | Pre-specified sensitivity analysis excluding 23 confirmed non-venomous species (Results 3.6). Multivariable logistic regression with three candidate predictors: D-dimer ≥620 ng/mL, MLW ≥13.5 min, platelet ≤226.5 ×10⁹/L (Results 3.5). |
| 18 | Intended sample size and how it was determined. | Post-hoc precision evaluation. With 33 HE-positive patients and observed AUC 0.874, Hanley–McNeil 95% CI half-width = 0.083 — acceptable for an exploratory pilot study. Methods Section 2.5. |
| **RESULTS — Participants** | | |
| 19 | Flow of participants, using a diagram. | Figure 1 (STARD flow diagram). See below.  225 patients screened for eligibility.  129 excluded (see Methods Section 2.2 for exclusion criteria).  96 patients enrolled and included in the primary analysis.  1 patient excluded from D-dimer analysis only (insufficient sample volume).  D-dimer analysis: HE n=33; non-HE n=62 (n=95 total).  All 96 patients included in HE reference standard adjudication. |
| 20 | Baseline demographic and clinical characteristics of participants. | Table 1. Age (mean ± SD), sex, time to ED (median [IQR]), snake identification, species breakdown (RVV, HNPV, cobra, krait, Malabar pit viper, non-venomous, unidentified), antivenom administered, local tissue damage — stratified by HE status with p-values. |
| 21a | Distribution of severity in those with the target condition. | Results Section 3.2 and Table 2. HE criteria met: haemotoxic bleeding 90.9%, MLW ≥15 min 48.5%, fibrinogen <100 mg/dL 36.4%, failed 20WBCT 30.3%, INR ≥1.4 30.3%, platelet <100×10⁹/L 9.1%. Antivenom in 84.8%. Delayed coagulopathy in 4 patients (2 HNPV, 2 RVV). |
| 21b | Distribution of alternative diagnoses in those without the target condition. | Table 1 and Results Section 3.1. Non-HE patients: non-venomous species (n=23), cobra (n=3), krait (n=3), Malabar pit viper (n=1), RVV without HE (n=4), HNPV without HE (n=6), unidentified without HE (n=23). |
| 22 | Time interval and clinical interventions between index test and reference standard. | Index test within 30 minutes of ED arrival. Reference standard adjudicated at 24 hours (with 6-hour reassessment). Antivenom given to 11 non-HE patients before lab results were available; reported in Results 3.2. |
| **RESULTS — Test results** | | |
| 23 | Cross-tabulation of index test results by reference standard results. | Table 3. At 620 ng/mL (n=95): TP=29, FN=4, TN=49, FP=13. Results Section 3.4. |
| 24 | Estimates of diagnostic accuracy and precision (95% CIs). | Table 3. D-dimer: AUC 0.874 (0.791–0.957); sensitivity 87.9% (72.7–95.2%); specificity 79.0% (67.4–87.3%); PPV 69.0% (54.0–80.9%); NPV 92.5% (82.1–97.0%); Youden J 0.669. All comparator AUCs and thresholds also in Table 3. Results Section 3.4. |
| 25 | Any adverse events from performing the index test or reference standard. | None. Index test required standard venepuncture only. No adverse events from HE adjudication. |
| **DISCUSSION** | | |
| 26 | Study limitations, including sources of potential bias, statistical uncertainty, and generalisability. | Discussion Section 4.1 (Limitations):  (i) Small sample (n=33 HE) — wide sensitivity CI (72.7–95.2%); external validation required.  (ii) Single-centre; Kerala species profile may not generalise to other Indian regions.  (iii) Partial incorporation bias: MLW/20WBCT, fibrinogen, platelet are both HE criteria and comparators; D-dimer AUC unaffected.  (iv) Youden threshold (620 ng/mL) data-derived; may not transfer across assay platforms.  (v) Single time-point D-dimer; serial measurements may improve yield.  (vi) Multivariable model EPV=11; wide CI for OR (6.13–79.25) reflects statistical instability. |
| 27 | Implications for practice, including intended use and clinical role of the index test. | Discussion and Conclusions (Section 5). Proposed role: early rule-out (post-test probability ~8% when D-dimer <620 ng/mL). Sequential strategy: D-dimer → MLW/20WBCT. Not ready for implementation without external validation. |
| **OTHER INFORMATION** | | |
| 28 | Registration number and name of registry. | ClinicalTrials.gov: NCT04520282. |
| 29 | Where the full study protocol can be accessed. | ICMR Haemostatic Variables in Snakebite Study (HISS), Project No. 5/4/8-21/2021-NCD-11. Full protocol available on request from the corresponding author (Dr Siju V Abraham; sijuvabraham@jmmc.ac.in). |
| 30 | Sources of funding and other support; role of funders. | ICMR-HISS (Project No. 5/4/8-21/2021-NCD-11). The funding body had no role in study design, data collection, analysis, interpretation, or manuscript preparation. |

**Abbreviations:** AUC = area under the ROC curve; CI = confidence interval; DOAC = direct oral anticoagulant; ED = emergency department; EPV = events per variable; FEU = fibrinogen-equivalent units; HE = haemostatic envenomation; HNPV = hump-nosed pit viper; ICMR = Indian Council of Medical Research; INR = international normalised ratio; IQR = interquartile range; ISTH DIC = International Society on Thrombosis and Haemostasis DIC score; JMMC&RI = Jubilee Mission Medical College & Research Institute; LR = likelihood ratio; MLW = modified Lee–White clotting time; NPV = negative predictive value; OR = odds ratio; POC = point-of-care; PPV = positive predictive value; PT = prothrombin time; ROC = receiver operating characteristic; RVV = Russell’s viper; STARD = Standards for Reporting of Diagnostic Accuracy Studies; VICC = venom-induced consumption coagulopathy; 20WBCT = 20-minute whole-blood clotting test; aPTT = activated partial thromboplastin time.
